# Equity of digital self-management tools in adults with multiple long-term conditions: a scoping review protocol

**DOI:** 10.1136/bmjopen-2025-108435

**Published:** 2025-11-09

**Authors:** Heather Walker, Timothy Robbins, Austen El-Osta, Lucy Stirland, David Taylor, Andy Jones, Emily McBride, Asra Aslam

**Affiliations:** 1 School of Cardiovascular and Metabolic Health, University of Glasgow, Glasgow, UK; 2 University Hospitals Coventry and Warwickshire NHS Trust, Coventry, UK; 3 Self-Care Academic Research Unit (SCARU), Department of Primary Care and Public Health, Imperial College London, London, UK; 4 Division of Psychiatry, Centre for Clinical Brain Sciences, The University of Edinburgh, Edinburgh, UK; 5 Institute of Security Science and Technology, Imperial College London, London, England, UK; 6 Centre for Health Services Studies, University of Kent, Canterbury, UK; 7 School of Mental Health and Psychological Sciences, King’s College London, London, England, UK; 8 School of Information, Journalism and Communication, The University of Sheffield, Sheffield, England, UK

**Keywords:** Chronic Disease, Digital Technology, Health Equity, Multimorbidity, Self Care, Self-Management

## Abstract

**Abstract:**

**Introduction:**

Adults living with multiple long-term conditions (MLTC)—defined as the presence of two or more physical or mental health conditions—often face fragmented and complex care. Digital tools offer scalable self-management solutions but may exacerbate inequities due to the digital divide and other factors. The aim of this scoping review is to map and summarise the existing literature on digital self-management tools used in MLTC, with a particular focus on how equity of access is considered in their development, implementation and evaluation.

**Methods and analysis:**

Scoping review methodology will be based on the Joanna Briggs Institute guidance for scoping reviews and Arskey and O’Malley’s framework and will be reported in alignment with Preferred Reporting Items for Systematic Review and Meta-Analyses extension for Scoping Reviews. Comprehensive search terms based on ‘multimorbidity’, ‘digital tools’ and ‘self-management’ have been developed. Peer-reviewed publications will be identified using MEDLINE, Embase, Emcare, Scopus, CINAHL and PubMed. Two reviewers will independently screen titles and abstracts, with subsequent full text review also being performed in duplicate to ensure they meet the eligibility criteria. Discrepancies will be resolved by discussion with a third reviewer. Included studies will focus on digital tools for the self-management of MLTC in adults (≥18 years old) in any setting. Equity dimensions will include, but are not limited to, digital literacy, treatment burden, socioeconomic status, polypharmacy and access disparities.

**Ethics and dissemination:**

Ethical approval is not required for this scoping review. The results of the scoping review will be published in an open access, peer-reviewed journal for wider dissemination. Additionally, findings will contribute to topic guides and mapping of a research networking event with key stakeholders (including patient and public involvement and engagement members, clinicians, researchers and industry) in MLTC, around the same subject area.

**STRENGTHS AND LIMITATIONS OF THIS STUDY**

- This scoping review will follow the Preferred Reporting Items for Systematic Reviews and Meta-analyses extension for Scoping Reviews guidelines to ensure transparency and robustness.
- Conceptualisation of the scoping review has used the novel ‘Team Science’ approach, with a key patient/public representative who is part of the core team.
- A diverse patient and public involvement group with lived experience of multiple long-term conditions (MLTC) was involved in developing this protocol and associated scoping review materials to ensure the research is relevant to individuals and carers living with MLTC.
- There are no date or setting limitations for this review; the work is however limited to publications in the English language.

## Introduction

 The increasing global prevalence of multiple long-term conditions (MLTC)—the presence of two or more physical or mental health conditions^1^—has brought attention to the limitations of current condition-specific care models.^2^ MLTC presents a complex challenge that requires integrated, person-centred approaches to care and management. Self-care, which includes self-management, has been defined by the WHO as ‘the ability of individuals, families and communities to promote health, prevent disease, maintain health and cope with illness and disability with or without the support of a health worker’.^3^ Digital health tools, ranging from mobile applications to online self-management platforms, offer opportunities to support individuals self-managing MLTC, improving autonomy, health behaviours and quality of life. Despite the proliferation of such technologies, concerns persist about their accessibility and equity.^4^

Equity of access refers to ensuring that digital self-management tools for MLTC are accessible, usable and effective for all individuals. It goes beyond the availability of tools; it is about actively identifying and addressing the barriers that different populations or individuals face in accessing and benefiting from digital health interventions. Equity in digital health and MLTC is influenced by factors including socioeconomic status, digital literacy, health literacy, education, ethnicity, language barriers, treatment burden, polypharmacy and cultural factors.^5^ Emerging literature indicates that while digital self-management tools are often validated in populations with single chronic conditions (eg, diabetes, hypertension, asthma),^6^ their efficacy and accessibility in the context of MLTC remain underexplored.^7^ Equitable digital self-management tools for MLTC are therefore largely under-researched despite the increasing use of smartphone applications and the rapid adoption of telemedicine during and following the COVID-19 pandemic.^8 9^

Considering the growing reliance on digital health solutions, it is critically important to ensure that these tools do not unintentionally widen existing health inequalities. It is important to prioritise this because current digital innovations often overlook the specific needs and contexts of underserved populations. By focusing on equity of access, we seek to contribute to a landscape promoting fair opportunities for all individuals with MLTC to manage their health and reduce health inequalities.

Given the anticipated heterogeneity of interventions, populations and outcome measures, as well as the novelty and evolving nature of digital health for MLTC, the proposed scoping review methodology allows for broad mapping of available evidence, identification of research gaps and development of conceptual frameworks.^10 11^

A preliminary search of MEDLINE and the Cochrane Database of Systematic Reviews revealed a single scoping review that assessed digital self-management tools for MLTC^12^ but no existing scoping or systematic reviews that comprehensively assess the equity and/or accessibility of digital self-management tools for MLTC. This scoping review is crucial because it will map existing evidence, identify gaps and inform a much-needed framework for equitable digital health interventions a step no one has comprehensively undertaken so far. Addressing this gap is essential to ensure digital innovations do not inadvertently widen health disparities but instead serve as a tool for promoting equity in healthcare.

### Aims and objectives

The primary aim of this scoping review is to map the existing literature on digital self-management tools for MLTC and evaluate how equity considerations are embedded in their design, implementation and evaluation. Specifically, we seek to: (i) identify and map existing digital self-management tools that are relevant to the care and management of people living with MLTC, (ii) examine the extent to which equity considerations have been integrated into the design, assessment, implementation or use of these tools, using established equity frameworks as reference, (iii) analyse equity-related factors uniquely affecting people with MLTC such as treatment burden, polypharmacy and heterogeneity of conditions and symptoms and then how these are addressed by the tools and (iv) Assess the application of existing equity frameworks in the development and evaluation of digital self-management tools, highlighting best practices and areas for improvement.

## Methods and analysis

At the time of publication, initial search strategies were performed in the relevant databases and title and abstract screening was complete. Full text review, data extraction and analysis phases are currently in progress and are expected to be completed within the next 3 months.

A scoping review is ideally placed to explore this area and summarise existing knowledge and tools. Given the anticipated heterogeneity of interventions, populations and outcome measures—as well as the novelty and evolving nature of digital health for MLTC—this methodology allows for broad mapping of available evidence, identification of research gaps and development of conceptual frameworks.^10 11^

Scoping review methodology will be based on Joanna Briggs Institute guidance for scoping reviews, supported by Arskey and O’Malley’s original framework.^13 14^ The review will be conducted and prepared using the Preferred Reporting Items for Systematic Review and Meta-Analyses extension for Scoping Reviews (PRISMA-ScR)^15^ (online supplemental file 1).

A minimum of two reviewers will independently screen titles and abstracts, with subsequent full text review also being performed in duplicate to ensure they meet the eligibility criteria. Differences will be resolved by discussion with a third reviewer (HW: clinical researcher with expertise in MLTC).

### Inclusion criteria

#### Participants

The review will consider peer-reviewed publications that report on adults (≥18 years of age) with two or more long-term, physical or mental, health conditions. Studies focusing on single conditions will be excluded unless explicitly related to MLTC contexts.

#### Concept

Sources of evidence that report information relating to the development, assessment, use or implementation of digital tools or interventions designed for self-care/management, including mHealth (mobile health), eHealth (use of digital technologies for health), telehealth, apps (web applications/software), wearable technology and internet-based platforms.

#### Context

Publications relating to digital self-management tools for adults with MLTC in any setting (community, secondary care, remote). Studies from all geographic locations are eligible, though high-income countries are anticipated to dominate. Unpublished research and studies not reported in the English language will be excluded.

### Types of sources

This scoping review will consider all sources of peer-reviewed published evidence on digital self-management tools in adults with MLTC. Sources of evidence will include primary research studies, observational studies, qualitative studies, studies reporting secondary data analysis (ie, systematic reviews), guidelines and policy documents. Expert opinions, editorials and websites will be excluded. A decision to include only published peer-reviewed work in the review was taken to improve the quality and accuracy of included studies,^16^ particularly given that scoping review methodology does not typically include critical appraisal of quality. Any unpublished sources (eg, conference abstracts) will be followed up with authors to assess if any subsequent publications occurred that may meet the inclusion criteria.

### Information sources

A three-step search process will be used: (1) an initial limited search of MEDLINE was performed to refine search terms, (2) full electronic database search and (3) grey literature searching and hand searching references lists and citations of included sources to identify further studies for inclusion. The following electronic databases were systematically searched on 27 May 2025 from inclusion to present date: MEDLINE (Ovid), Embase (Ovid), Emcare (Ovid), CINAHL (EBSCO), Scopus and PubMed. English language restrictions were applied.

### Search strategy

Comprehensive search terms based on ‘multimorbidity’, ‘digital tools’ and ‘self-management’ (online supplemental file 2), combining MeSH (Medical Subject Headings) terms (and relevant synonyms) and the Boolean operators ‘AND’ and ‘OR’ for search strings have been developed. The search terms were initially developed in MEDLINE and then adapted for use in other databases. Search terms were developed by the research team (HW and TR) with support from Clinical Evidence Based Information Specialists and a specialist librarian.

### Study selection

Identified references will be imported into Covidence systematic review software (2025),^17^ where duplicates will be automatically removed. A team of reviewers will conduct title/abstract and full-text reviews, in duplicate as described earlier against the prespecified inclusion criteria. Any disagreements at either stage will be resolved through discussion or by consulting a third reviewer (HW). The screening process will be documented and presented using a PRISMA-ScR flow diagram. All screening will be conducted via Covidence.

### Data charting

A standardised electronic data extraction template will be developed, piloted and refined. Extracted data will include: author, year, country, setting, study design, population description, MLTC definition, intervention/tool description, modality of tool delivery, equity considerations and patient and public involvement and engagement (PPIE). Equity dimensions for consideration will include but are not limited to digital literacy, health literacy, language barriers, education, treatment burden, socioeconomic status, polypharmacy, cultural factors and access disparities. In line with the iterative process of a scoping review, the data extraction form will be updated and refined throughout the review, as necessary and in response to identified literature. All data charting will be performed within the Covidence system. Formal assessment of the methodological quality of included studies is not necessary, as the focus of the scoping review is to assess and map existing knowledge and any gaps in this area.

### Data analysis and summary

Following the data charting process, identified digital self-management tools for MLTC will be presented in narrative and tabular form. Results including the characteristics of included tools and studies will be summarised descriptively. Equity-related findings will be presented using appropriate existing equity frameworks such as WHO Health Equity Assessment Toolkit^18^ or framework for digital health equity produced by Richardson *et al*.^19^ However, given the anticipated diversity of included studies and assessment of equity these will not be predefined.

### Novel team science approach

One of the key strengths of our proposed methodology is the explicit and early adoption of a *Team Science* approach, embedded from the outset of our collaboration at the National Institute for Health and Care Research (NIHR) Team Science event and further developed since. While increasingly recognised in the literature,^20^ Team Science is rarely applied so deliberately during early methodological planning. Traditional research models often form collaborations post hoc, relying on hierarchical or discipline-bound structures that can limit innovation.

In contrast, we have operationalised Team Science as a core methodological feature, using a Collaborative Planning Approach to co-develop a Collaboration Plan that guides team functioning—covering communication, leadership, conflict resolution and inclusivity. This structured model reflects our commitment to mutual respect, equitable contribution and iterative learning, and stands apart from conventional models by prioritising team dynamics as much as scientific aims. We believe Team Science methodology positions us to deliver more innovative, inclusive and impactful research in MLTC.

## Ethics and dissemination

This scoping review does not require ethical approval. The findings of the review will be shared through professional networks, national conferences and publication in an open access peer-reviewed journal. All data will be managed within the Covidence system.

### Patient and public involvement and engagement

The conceptualisation of the scoping review has used the novel ‘Team Science’” approach described earlier, with a key patient/public representative (DT) who is part of the core team. Additionally, the scoping review and associated materials have been developed with a diverse patient and public involvement group with lived experience of MLTC. The findings of this review will be used to shape stakeholder workshops to ultimately develop a framework for equitable digital self-management tool.

## Supplementary material

10.1136/bmjopen-2025-108435 online supplemental file 1

## Data Availability

Data sharing is not applicable as no datasets were generated and/or analysed for this study.
