# Supplementary material for: Equity of digital self-management tools in adults with multiple long-term conditions: a scoping review protocol
Source: BMJ Open. 2025 Nov 9;15(11):e108435. doi: 10.1136/bmjopen-2025-108435 (PMC12598953; doi:10.1136/bmjopen-2025-108435)
Supplement: Supplementary file 2 [file bmjopen-2025-108435.reviewer_comments.pdf]

PEER REVIEW HISTORY

BMJ Open publishes all reviews undertaken for accepted manuscripts. Reviewers are asked to complete a checklist review form and are provided with free text boxes to elaborate on their assessment. These free text comments are reproduced below.

ARTICLE DETAILS

Title (Provisional)

Equity of digital self-management tools in adults with multiple long-term conditions: a scoping review protocol

Authors

Walker, Heather; Robbins, Timothy; El-Osta, Austen; Stirland, Lucy; Taylor, David; Jones, Andy; McBride, Emily; Aslam, Asra

---

VERSION 1 - REVIEW

**Reviewer**	**1**
**Name**	**Seakamela, Kagiso P**
**Affiliation**	**University of Limpopo**
**Date**	**23-Sep-2025**
**COI**	**None**

I did not see any checklist in the supplement, except for the search strategy.

VERSION 1 - AUTHOR RESPONSE

Reviewer: 1
Mr. Kagiso P Seakamela, University of Limpopo
Comments to the Author:
I did not see any checklist in the supplement, except for the search strategy.

As above we have now included the PRISMA-ScR checklist in the supplementary materials.
